# Weight change following knee and hip joint arthroplasty–a six-month prospective study of adults with osteoarthritis

**DOI:** 10.1186/s12891-015-0598-y

**Published:** 2015-06-07

**Authors:** Andrew J Teichtahl, Emma Quirk, Paula Harding, Anne E Holland, Clare Delany, Rana S Hinman, Anita E Wluka, Susan M Liew, Flavia M Cicuttini

**Affiliations:** Department of Epidemiology and Preventive Medicine, School of Public Health and Preventive Medicine, Monash University, Alfred Hospital, Melbourne, VIC 3004 Australia; Baker IDI Heart and Diabetes Institute, Commercial Road, Melbourne, VIC 3004 Australia; Department of Physiotherapy, Alfred Hospital, Melbourne, VIC 3004 Australia; Department of Physiotherapy, Centre for Health, Exercise and Sports Medicine, School of Health Sciences, University of Melbourne, Victoria, Australia; Department of Physiotherapy, La Trobe University, Melbourne, Vic Australia; School of Health Sciences, University of Melbourne, Victoria, Australia; Department of Orthopaedic Surgery, Alfred Hospital, Melbourne, VIC 3004 Australia; Department of Surgery, Monash University, Melbourne, Australia

**Keywords:** Osteoarthritis, Hip, Knee, Joint replacement, Obesity, Weight loss

## Abstract

**Background:**

Inconsistent findings of weight change following total knee (TKA) and hip (THA) arthroplasty may largely be attributable to heterogeneous cohorts and varied definitions of weight loss. This study examined weight change following TKA and THA for osteoarthritis (OA).

**Methods:**

64 participants with hip or knee OA were recruited from orthopaedic joint arthroplasty waiting lists at a single major Australian public hospital between March and October 2011. The Short Form (SF) 12 survey was used to assess baseline physical and mental functioning. 49 participants completed 6 month follow-up (20 from the THA group and 29 from the TKA group).

**Results:**

The majority of subjects lost weight (>0 kg) 6 months following THA (70 %) and TKA (58.6 %). When at least a 5 % reduction in total body weight was used to define clinically significant weight loss, the proportion of people with weight loss was 37.9 % for TKA and 25 % for THA. Greater weight loss occurred 6 months following TKA compared with THA (7.2 % versus 3.7 % of body weight; *p* = 0.04). Worse pre-operative physical functioning (SF-12) was associated with greater weight loss following TKA (β = 0.22 kg, 95 % CI 0.02-0.42 kg; *p* = 0.04).

**Conclusion:**

Most people lost weight (>0 kg) 6 months following TKA and THA and a considerable proportion of people achieved ≥5 % loss of body weight. The magnitude of weight loss was greater following TKA than THA, with worse pre-operative function being a predictor of more weight loss. Further attention to weight management is required to assist a greater number of people to achieve a larger magnitude of weight loss following knee and hip joint arthroplasty.

## Key messages

- Inconsistent findings of weight change following total knee (TKA) and hip (THA) arthroplasty may largely be attributable to heterogeneous cohorts and varied definitions of weight loss.
- We confirm that the majority of people achieve some weight loss following TKA or THA, with the magnitude being greater following TKA.
- Poorer pre-operative physical functioning helps to predict individuals who will lose more weight following TKA.

## Background

Total knee (TKA) and hip (THA) arthroplasty are commonly performed for the management of painful end-stage osteoarthritis (OA). Although patient satisfaction rates have been shown to be approximately 90 % following TKA or THA for OA in the Australian public healthcare system [1], studies examining post-operative outcomes have focused on pain, joint function and activity limitations. However despite only minimal, if any, post-arthroplasty improvement in physical activity levels [2, 3], patients expect to lose weight [4]. Obesity is a recognised risk factor for both end-stage hip and knee OA, and it is therefore important to determine whether arthroplasty augments weight-management. In a global context, the recent 11^th^ annual report from the United Kingdom Joint Registry, which examined surgical outcomes, focused mainly on mortality and revision rates [5]. The paucity of data concerning weight loss post-arthroplasty is however surprising, given that as little as 1 % loss of weight results in a reduction in the rate of cartilage volume loss and an improvement in knee pain, stiffness and function [6], which may have implications for the contralateral knee and other joints. A recent systematic review was unable to make any conclusions about weight or body composition changes after joint arthroplasty [7].

There are a number of reasons for the inconsistent results among the few studies examining post-operative weight change. A major issue is the heterogeneity of the examined cohorts. For instance, while some studies included people with both inflammatory arthritides and OA [8–12], other studies pooled results from TKA and THA [9]. Outcome methods have also varied. Whereas some studies have employed “any weight loss” as an outcome [8, 13–15], other studies have used at least 5 % body weight to signify clinically meaningful weight loss [9, 11, 12, 16, 17]. However, a commonality among these studies is that there are a proportion of people who gain and lose weight, while others remain weight neutral. This raises the possibility of identifying key characteristics that could stratify the likelihood of post-arthroplasty weight loss.

The aim of this prospective study was to i) examine weight change in people following total knee and hip joint arthroplasty for OA and ii) to determine factors that may be associated with post-arthroplasty weight loss.

## Methods

### Data collection

This study was a prospective case series that recruited participants between March 1 and October 15, 2011, from the Alfred, a major tertiary referral public hospital in Melbourne, Australia.

Inclusion criteria were age between 50 and 80 years (inclusive), a diagnosis of hip or knee OA, and waitlisting for a primary THA or TKA. Only participants undergoing their first (primary) joint replacement were included. Participants who were in high-level care facilities (such as nursing homes) or were requiring a wheel-chair were excluded. A lower age cut-off of 50 was chosen in an attempt to reduce the potential for non-OA indications for surgery (e.g. inflammatory arthritides such as ankylosing spondylitis). Participants with comorbidities that significantly affected their level of physical activity such as a previous stroke and those with English language or cognitive barriers were also excluded. A total of 141 adults waitlisted for an elective THA or TKA attended the preadmission clinic between March and October 2011. Seventy-seven adults met the eligibility criteria and 64 people were recruited for the study. Of these, 49 completed 6-month follow-up. Participants completing follow-up were no different in relation to their baseline demographics (age, gender and BMI) than those who had not completed the study (data not shown). Written informed consent was obtained.

The type of prosthesis used for surgery was chosen by the individual orthopaedic consultant but was limited at the femoral side to either the Corail ® Total Hip System (DePuy Orthopaedics Inc., Warsaw, IN, USA) (for uncemented THA) or the Exeter Hip (Stryker Orthopaedics, Mahwah, NJ, USA) (for cemented THA). A Pinnacle or Duraloc ® System (DePuy Orthopaedics Inc), or a Trident (Stryker Orthopaedics) was available for the acetabular component. The GENESIS II (Smith & Nephew Inc, Memphis, TN USA) or Stryker ® Triathlon (Stryker Orthopaedics) prosthesis was used for all TKAs. All participants were managed with the same postoperative protocol and were permitted to weightbear as tolerated. During the inpatient post-operative phase, routine post-operative care included daily physiotherapy (mobilization day 1 post-operatively) and a bed-based exercise program. Sixty-one percent of participants were discharged home with ongoing outpatient physiotherapy provided by community services. The remaining participants were discharged to inpatient rehabilitation centres.

Preoperative data were collected at the preadmission clinic by an allied health assistant. Followup data were collected at the routine 6-month postoperative review appointment by an orthopaedic outpatient physiotherapist. Baseline participant information pertaining to age, sex, comorbidities, height, weight, and body mass index (BMI) was extracted from the anaesthetic assessment conducted at the preadmission clinic and recorded in the medical history. Comorbidities were categorized as cardiovascular, diabetes, respiratory, musculoskeletal (excluding the affected hip or knee), or other. Since participants could have more than one comorbidity in each category, the total number of comorbidities was also recorded. The Short Form (SF) 12 version 1, which contains twelve questions regarding physical and emotional well-being to determine a physical component and mental component summary score (PCS and MCS respectively), was also collected at baseline [18]. A lower SF-12 score indicated poorer physical or mental health.

At the 6 month postoperative review, weight was measured to the nearest 0.1 kg (shoes, socks, and bulky clothing removed) using a single pair of electronic scales. Height was measured to the nearest 0.1 cm (shoes and socks removed) using a stadiometer. From these data, body mass index [BMI (kg m^−2^)] was calculated. At followup information from the medical file regarding length of stay in hospital and adverse events was recorded.

This study was approved by the Alfred Hospital Research Ethics Committee.

### Statistical analyses

Independent t-tests were used to compare unadjusted differences in the characteristics between subjects undergoing TKA and THA. General linear models were used and estimated marginal means (EMMs) were created, adjusting for age, gender and the number of comorbidities to determine whether the magnitude of weight loss differed between people following TKA or THA. The primary outcome measures were change in BMI, weight (kg) and percentage weight. Based on previous data [13] we estimated a sample size of approximately 26 participants in each group would enable 80 % power to detect statistically significant differences in weight (kg) change among people undergoing TKA and THA (*p* = 0.05, two-tailed). Linear regression analyses were used to examine the relationships between baseline variables and weight change following joint arthroplasty. A *p*-value of less than 0.05 (two-tailed) was considered statistically significant. All analyses were performed using SPSS statistical package (standard version 21.1 SPSS, Chicago, IL, USA).

## Results

Twenty subjects who underwent THA and 29 subjects who underwent TKA completed follow-up (Table 1). There were no significant differences in the baseline characteristics between people who completed follow-up (*n* = 49) and those who did not (*n* = 64) (data not shown). Reasons for loss to follow-up included declining surgery (*n* = 8) and non-attendance at follow-up (*n* = 7). At baseline, 9 (45 %) people having THA were overweight (BMI ≥ 25–30 kgm^−2^), while 8 (40 %) were obese or heavier (BMI ≥ 30 kgm^−2^). Ten (34 %) people proceeding to TKA were overweight (BMI ≥ 25–30 kgm^−2^) while 16 (55 %) were obese or heavier (BMI ≥ 30 kgm^−2^). The pooled cohort had a mean BMI of 30.6 ± 5.9 kgm^−2^.

**Table 1:** Subject characteristics

	THA	TKA	*P*
	(*n* = 20)	(*n* = 29)	
Baseline			
Age (years)	69.5 (8.8)	67.4 (8.3)	0.41
Gender (% female)	65 %	65.5 %	0.97*
Weight (kg)	79.4 (19.2)	83.8 (19.2)	0.43
Height (m)	1.64 (0.11)	1.63 (0.11)	0.76
BMI (kg m^−2^)	29.2 (4.9)	31.5 (6.3)	0.16
Length of stay (days)	6.0 (1.8)	7.1 (5.6)	0.31
PCS	28.9 (5.8)	31.7 (8.4)	0.18
MCS	39.2 (13.6)	45.1 (12.8)	0.13
Co-morbidities (%)			
Cardiovascular disease	65	55	0.58*
Diabetes mellitus	20	21	0.98*
Respiratory	15	10	0.61*
Musculoskeletal	35	34	0.87*
Average number of comorbidities	2.9 (2.1)	2.4 (1.5)	0.35
Follow-up			
BMI (kg m^−2^)	28.6 (5.0)	30.5 (6.4)	0.25
Weight (kg)	77.5 (19.2)	81.0 (18.9)	0.54
Change			
BMI (kg m^−2^)	−0.68 (1.08)	−1.07 (1.80)	0.35
Weight (kg)	−1.84 (2.95)	−2.86 (4.82)	0.36
Weight (%)	−2.4 (4.0)	−3.3 (5.7)	0.51
Weight loss			
Number of people who lost weight n (%)	14 (70)	17 (58.6)	0.42*
BMI reduction (kg m^−2^)	−1.1 (0.2)	−2.2 (0.4)	0.02
Weight (kg)	−3.09 (2.57)	−5.93 (3.92)	0.02
Weight (%)	−4.1 (3.4)	−6.9 (4.7)	0.06
>5 % body weight reduction, n, (%)	5 (25)	11 (37.9)	0.34*
Weight gain			
Number of people who gained weight n (%)	4 (20)	10 (34.5)	0.27*
BMI increase (kg m^−2^)	0.6 (0.3)	0.7 (0.3)	0.48
Weight (kg)	1.7 (0.8)	1.8 (1.1)	0.66
Weight (%)	2.4 (1.5)	2.3 (1.6)	0.37
>5 % body weight gain, n, (%)	0 (0)	1 (10)	0.51*

Unless otherwise stated, results displayed as mean (standard deviation)

*P*-values derived from independent t-tests (excluding * which indicates Chi-square analyses)

PCS/MCS – physical/metal components score from Short-Form 12 survey

Seventy percent of subjects who had a THA, and 58.6 % of subjects who had a TKA lost weight (>0 kg), although this was reduced to 25 % and 37.9 % respectively when a 5 % threshold of weight loss was required. Using the 5 % weight change criterion, only one subject (<2 % of the cohort) gained weight, although 34.5 % of TKA patients gained weight (>0 kg), while 20 % of THA patients gained weight (>0 kg). Significantly more weight loss occurred 6 months following TKA compared with THA (5.9 kg versus 3.1 kg; *p* = 0.02). Post-operative complications for participants completing 6 month follow-up are shown in Table 2.

**Table 2:** Post-operative complications for people completing follow-up (Within 6 months of arthroplasty)

	THA	TKA
	(*n* = 20)	(*n* = 29)
Mortality	0 (0.0)	0 (0.0)
Deep vein thrombosis	1 (5.0)	0 (0.0)
Post-operative hypotension	1 (5.0)	1 (3.4)
Wound ooze	4 (20.0)	2 (6.8)
Septic arthritis	0 (0.0)	1 (3.4)
Haematoma	1 (5.0)	1 (3.4)
Acute renal failure	0 (0.0)	1 (3.4)
Inpatient fall	0 (0.0)	1 (3.4)
Manipulation under anaesthetic	0 (0.0)	4 (13.8)

Results represented by frequency (%)

Estimated marginal means for the amount of weight loss are presented in Table 3 After adjusting for baseline age, gender, weight and number of co-morbidities, there was a significantly greater reduction in measures of obesity following TKA compared with THA: BMI (2.3 kg m^−2^ versus 1.2 kg m^−2^; *p* = 0.03), weight (5.8 kg versus 3.2 kg; *p* = 0.05) and percentage body weight (7.2 % versus 3.7 %; *p* = 0.04).

**Table 3:** Estimated marginal means for weight loss

	Crude (SEM)			Adjusted (SEM)^a^		
	THA	TKA	*P*	THA	TKA	*P*
	(*n* = 14)	(*n* = 17)		(*n* = 14)	(*n* = 17)	
BMI (kg m^−2^)	1.1 (0.5)	2.2 (0.5)	0.02	1.1 (0.3)	2.3 (0.3)	0.03
Weight (kg)	3.1 (0.9)	5.9 (0.8)	0.02	3.2 (0.9)	5.8 (0.8)	0.05
Weight (%)	4.1 (1.1)	6.9 (1.0)	0.06	3.7 (1.1)	7.2 (1.0)	0.04

^a^Estimated marginal means adjusted for baseline age, gender, weight and number of comorbidities

The relationships between baseline characteristics and post-operative weight loss are shown in Table 4. After adjusting for potential confounders, pre-operative physical functioning (SF-12) was associated with weight change following TKA (β = 0.22 kg, 95 % CI 0.02-0.42 kg; *p* = 0.04). That is, for every 1 point worsening score in baseline physical functioning, there was 0.22 kg weight loss following TKA. No examined variables were associated with weight loss following THA.

**Table 4:** Baseline variables and post-arthroplasty weight change (Kg)

	THA		TKA	
	Multivariate*	*P*	Multivariate*	*P*
	β (95 % CI)		β (95 % CI)	
Age	−0.06 (−0.29, 0.18)	0.62	−0.08 (−0.32, 0.16)	0.50
Gender	−0.49 (−5.1, 4.1)	0.82	−3.45 (−7.54, 0.65)	0.10
Weight	−0.02 (−0.13, 0.09)	0.70	−0.10 (−0.20, 0.01)	0.07
Number of comorbidities	0.03 (−0.92, 0.97)	0.95	−0.76 (−2.04, 0.53)	0.24
PCS	0.14 (−0.29, 0.57)	0.51	0.22 (0.02, 0.42)	0.04
MCS	−0.02 (−0.17, 0.14)	0.81	−0.08 (−0.25, 0.09)	0.35

*Adjusted for baseline age, gender, weight and number of comorbidities

PCS/MCS – physical/mental components score from Short-Form 12 survey

## Discussion

This study has demonstrated that the majority of subjects lose weight following TKA and THA, although the magnitude of weight loss is significantly greater following TKA. Worse pre-operative physical functioning was associated with greater weight loss following TKA.

A recent systematic review was unable to conclude whether or not weight loss is a common post-arthroplasty occurrence [7]. A major reason for the inconsistent results among past studies is related to the heterogeneity of the populations studied and the outcome measures employed. For instance, while some studies included people with both inflammatory arthritides and OA [8–12], other studies pooled results from TKA and THA [9]. The current study has selected people undergoing TKA and THA for OA and analysed these groups separately. We confirm that the majority of people lost weight (>0 kg) 6 months post-operatively (58.6 % for TKA and 70 % for THA). Although a more stringent cut-off for weight loss (≥5 % body weight) reduced the number of people achieving this target, a considerable proportion of people (37.9 % following TKA and 25 % following THA) were still able to achieve this threshold. Taken together, these results suggest that while the majority of people achieve some weight loss following either TKA or THA, further attention to weight management in the post-operative period may assist a greater number of people achieve a larger magnitude of weight loss following knee and hip joint arthroplasty. Using the 5 % weight change criterion, only one subject (<2 % of the cohort) gained weight, although 34.5 % of TKA patients gained weight (>0 kg), while 20 % of THA patients gained weight (>0 kg).

In this study, we have demonstrated that the magnitude of weight loss is significantly greater following TKA compared with THA. This is an important distinction, given that there is evidence that metabolic syndrome is more common with knee rather than hip OA [19], while obesity is also associated with the need for bilateral TKA in OA [20, 21]. In this study, the BMI of people with end-stage knee OA requiring joint arthroplasty was greater than that for hip OA, although the difference was not statistically significant. Weight loss imparted by TKA may therefore be a potential means of reducing the burden of the metabolic syndrome, and may even help to manage possible co-existing disease in the contralateral knee. We have shown that even as little as 1 % loss of weight results in a reduction in the rate of cartilage volume loss and an improvement in knee pain, stiffness and function [6], so that the effect of weight-loss imparted by surgery may be beneficial to the non-operated native knee. These hypotheses do however require substantiation in future studies. Moreover, this is the first study to examine and identify a determinant of weight loss following arthroplasty. Previously it had been demonstrated that patients with worse pre-operative function had the greatest outcomes following TKA, as determined by a composite physical function score [22]. Similarly, the current study found that people with worse physical function preceding TKA had the greatest weight loss post-operatively. This may have occurred because the intervention (TKA) improved activity levels, subsequently promoting greater energy expenditure and weight loss. Nevertheless, such claims require substantiation and it has been shown that TKA at best, only modestly improves physical activity levels [2]. Despite our cohort self-reporting greater physical activity post THA or TKA, no objective increase in physical activity was demonstrable post-operatively [3]. Regardless of the mechanism, these data support the possibility of identifying a subgroup of people who may, as an adjunct to having their end-stage OA treated by arthroplasty, also derive weight management benefits.

Despite this prospective study being limited by its modest sample size, we had adequate power to demonstrate between group differences in weight-loss. This was a single-center study conducted in a public hospital over a 6 month follow-up duration. Although this was a strength of the study since no potential confounding from different acute and sub-acute protocols across multiple care-settings was introduced, multi-center studies examining outcomes in both public and private hospitals over longer durations are required to substantiate the generalizability of this study’s findings. Moreover, we have used crude measures for assessing changes in weight. Given forced immobility can occur in the immediate post-operative setting, body composition studies would help to determine whether fat or fat free mass accounts for the loss in body mass following joint arthroplasty. Nevertheless, our follow-up of 6 months is likely to have been sufficient time for rehabilitation and conditioning to have reversed any acute loss of fat free mass secondary to deconditioning. We advocate the need for body composition studies to further examine this concept. Moreover, we acknowledge that physical, mental and social factors all influence weight change in both the pre and post-operative period. A better understanding of how these biopsychosocial factors influence weight management in orthopaedic settings may assist patients to achieve weight loss.

## Conclusions

Most people lost weight (>0 kg) 6 months following TKA and THA and a considerable proportion of people achieved ≥5 % loss of body weight. The magnitude of weight loss was greater following TKA than THA, with worse pre-operative function being a predictor of more weight loss. Further attention to weight management is required to assist a greater number of people to achieve a larger magnitude of weight loss following knee and hip joint arthroplasty.

## Abbreviations

BMI: Body mass index
EMM: Estimated marginal mean
MCS: Mental component score
OA: Osteoarthritis
PCS: Physical component score
SF: Short form
TKA: Total knee arthroplasty
THA: Total hip arthroplasty
